# Principles of Zoology: Touching the Structure, Development, Distribution and Natural Arrangement of the Races of Animals, Living and Extinct; with Illustration

**Published:** 1849-01

**Authors:** 


Art. V.—*Principles of Zoölogy: Touching the Structure, Development, Distribution and Natural Arrangement of the Races of Animals, living and extinct;* with numerous illustrations. For the use of Schools and Colleges. Part I. *Comparative Physiology.* By Louis Agassiz and Augustus A. Gould. Boston: Gould, Kendall & Lincoln. 1848. 12mo. pp. 207.

We wish to call the attention of our readers to the work, the title of which we have given above, and to the series of which it is the beginning. The names of its authors are an ample warrant for the accuracy and ability with which the task will be executed; and as to the interest of the subject, we suppose there can be but one opinion among educated men. Although the work before us is designed especially for "schools and colleges," it is one in which most readers will find much that is new to them; and the chapters on the geographical distribution of animals, and their distribution in time, will reward the attention of every class of readers. The characteristic feature of the work is, that while it is elementary, it is profound—that it is adapted to the capacity of the beginner, and yet is capable of interesting and instructing all but adepts in the science of which it treats.

We feel that we should be rendering a high service to our profession, if we could foster and stimulate among its young men a taste for the study of natural history. How intimately it is associated with medicine, and how happily it exercises and expands the faculties of the mind! It is a perpetual feast, to which the physician, wearied and worn out by the toils and anxieties of his arduous calling, may address himself with ever new delight. However other studies may exhaust, this never cloys or fatigues. Once entered upon, and a taste for it once contracted, it is sure to be pursued with increasing zeal, and with a reward felt by the curious and cultivated mind to be the one most above price.

The following is the beautiful conclusion of this admirable little work;

"From the above sketch it is evident that there is a manifest progress in the succession of beings on the surface of the earth. This progress consists in an increasing similarity to the living fauna, and among the Vertebrates, especially, in their increasing resemblance to Man.

"But this connection is not the consequence of a direct lineage between the faunas of different ages. There is nothing like parental descent connecting them. The Fishes of the Paleozoic age are in no respect the ancestors of the Reptiles of the Secondary age nor does Man descend from the Mammals which preceded him in the Tertiary age. The link by which they are connected is of a higher and immaterial nature; and their connection is to be sought in the view of the Creator himself, whose aim, in forming the earth, in allowing it to undergo the successive changes which Geology has pointed out, and in creating successively all the different types of animals which have passed away, was to introduce Man upon the surface of our Globe. Man is the end towards which all the animal creation has tended, from the first appearance of the first Paleozoic Fishes.

"In the beginning His plan was formed, and from it He has never swerved in any particular. The same Being who in view of man's moral wants, provided and declared, thousands of years in advance, that "the seed of the woman shall bruise the serpent's head," laid up also for him in the bowels of the earth, those vast stores of granite, marble, coal, salt, and the various metals, the products of its several revolutions; and thus was an inexhaustible provision made for his necessities, and for the development of his genius, ages in anticipation of his appearance.

"To study, in this view, the succession of animals in time, and their distribution in space, is therefore to become acquainted with the ideas of God himself. Now, if the succession of created beings on the surface of the globe is the realization of an infinitely wise plan, it follows that there must be a necessary relation between the races of animals, and the epoch at which they appear. It is necessary therefore, in order to comprehend Creation, that we combine the study of extinct species with that of those now living. since one is the natural complement of the other. A system of Zoölogy will consequently be true, in proportion as it corresponds with the order of succession among animals."

